# Neural correlates of short-term memory in primate auditory cortex

**DOI:** 10.3389/fnins.2014.00250

**Published:** 2014-08-14

**Authors:** James Bigelow, Breein Rossi, Amy Poremba

**Affiliations:** Department of Psychology, University of IowaIowa City, IA, USA

**Keywords:** *Macaca mulatta*, working memory, A1, primary auditory cortex, rhesus macaque, recognition memory

## Abstract

Behaviorally-relevant sounds such as conspecific vocalizations are often available for only a brief amount of time; thus, goal-directed behavior frequently depends on auditory short-term memory (STM). Despite its ecological significance, the neural processes underlying auditory STM remain poorly understood. To investigate the role of the auditory cortex in STM, single- and multi-unit activity was recorded from the primary auditory cortex (A1) of two monkeys performing an auditory STM task using simple and complex sounds. Each trial consisted of a sample and test stimulus separated by a 5-s retention interval. A brief wait period followed the test stimulus, after which subjects pressed a button if the sounds were identical (match trials) or withheld button presses if they were different (non-match trials). A number of units exhibited significant changes in firing rate for portions of the retention interval, although these changes were rarely sustained. Instead, they were most frequently observed during the early and late portions of the retention interval, with inhibition being observed more frequently than excitation. At the population level, responses elicited on match trials were briefly suppressed early in the sound period relative to non-match trials. However, during the latter portion of the sound, firing rates increased significantly for match trials and remained elevated throughout the wait period. Related patterns of activity were observed in prior experiments from our lab in the dorsal temporal pole (dTP) and prefrontal cortex (PFC) of the same animals. The data suggest that early match suppression occurs in both A1 and the dTP, whereas later match enhancement occurs first in the PFC, followed by A1 and later in dTP. Because match enhancement occurs first in the PFC, we speculate that enhancement observed in A1 and dTP may reflect top–down feedback. Overall, our findings suggest that A1 forms part of the larger neural system recruited during auditory STM.

## Introduction

One of the vital cognitive processes enabling adaptive behaviors in humans and other animals is short-term memory (STM), i.e., the temporary retention of behaviorally-relevant information in the absence of direct stimulation (Goldman-Rakic, 1995). In contrast to the sizable literature describing visual STM and its neural substrates, relatively few studies have investigated auditory STM at the behavioral or neuronal levels. This central function of the auditory system is fundamental to vital behaviors such as conspecific communication. Thus, one of the remaining steps toward a complete view of the functional organization of the auditory system is a more detailed understanding of auditory STM and its underlying neural processes.

Early studies investigating the neural substrates of visual STM in non-human primates singled out the critical involvement of the lateral prefrontal cortex (PFC) in tasks that included a memory delay. Thus, bilateral lesions of the PFC produced severe performance deficits in canonical tests of STM such as delayed response and delayed matching-to-sample (DMS; Jacobsen, 1935; Mishkin and Manning, 1978; Goldman-Rakic, 1987). Further, electrophysiological studies have shown that neuronal activity in the PFC changes in ways that correspond to STM task demands. For example, many studies have reported that significant proportions of PFC neurons exhibit sustained changes in firing rates (often elevated but sometimes suppressed) during the retention phase of STM tasks (e.g., Fuster and Alexander, 1971; Miller et al., 1996; Shafi et al., 2007). Moreover, when task contingencies require the subject to identify whether a test stimulus matches a prior sample stimulus, many PFC neurons exhibit significantly enhanced firing rates when a match is detected, whereas other cells exhibit significant match suppression (e.g., Miller et al., 1996).

Although additional research has largely validated the prominent role of the PFC in STM, growing evidence has required expanded models of STM, which accommodate the involvement of earlier sensory cortical areas (Constantinidis and Procyk, 2004; Pasternak and Greenlee, 2005; Postle, 2006). For non-spatial forms of visual STM, this includes coactivation and functional interactions between PFC and visual areas in the temporal lobes (Fuster and Jervey, 1981, 1982; Fuster et al., 1985; Miller et al., 1993, 1996; Miller and Desimone, 1994; Constantinidis and Procyk, 2004; Ranganath, 2006), whereas spatial forms of visual STM rely heavily upon fronto-parietal interaction (Friedman and Goldman-Rakic, 1994; Chafee and Goldman-Rakic, 1998, 2000; Quintana and Fuster, 1999; Curtis, 2006; Klingberg, 2006). Further, correlates of visual STM have been observed in early visual areas in the occipital lobe including primary visual cortex (Supèr et al., 2001; Sligte et al., 2009; Emrich et al., 2013), as well as in the mediodorsal nucleus of the thalamus (Schulman, 1964; Fuster and Alexander, 1973; Isseroff et al., 1982). Thus, contemporary views hold that visual STM is enabled by collaborations among multiple nodes of a widespread network comprising cortical and subcortical structures. Within this system, the roles of the PFC include integrating sensory inputs, selecting task-relevant information, and exerting top–down influence on earlier sensory areas, thus modulating responses to behaviorally-relevant stimuli and ultimately guiding goal-directed behavior (Miller and Cohen, 2001; Fuster, 2008).

Fewer studies have investigated the neural substrates of auditory STM, perhaps in part due to the difficulties associated with training non-human primates to perform auditory tasks (Cohen et al., 2005; Fritz et al., 2005; Munoz-Lopez et al., 2010; Scott et al., 2012; Bigelow and Poremba, 2014). Nevertheless, the available evidence suggests that neural circuits underlying auditory and visual STM share at least some of the same organizational and functional principles (Poremba and Bigelow, 2013). Some of the earliest attempts to characterize the role of the PFC in auditory STM used delayed response or DMS tasks in which subjects were trained to match an auditory sample to a visual test. In both spatial and non-spatial versions of these tasks, neurons in the PFC exhibited changes in firing rate during the retention interval similar to those observed in visual tasks (Joseph and Barone, 1987; Bodner et al., 1996; Fuster et al., 2000). Correspondingly, performance in these tasks was significantly impaired by PFC lesions or cooling inactivations (Blum, 1952; Sierra-Paredes and Fuster, 2002). Subsequent studies have also observed neurophysiological correlates of audiospatial STM in the PFC using purely auditory delayed response and DMS tasks (Kikuchi-Yorioka and Sawaguchi, 2000; Artchakov et al., 2007, 2009). Outside of the PFC, several lesion and recording studies have indicated that auditory areas in the temporal lobe are important for non-spatial auditory STM (Colombo et al., 1990, 1996; Fritz et al., 2005; Ng et al., 2014), and one study has indicated the involvement of the lateral intraparietal area in spatial auditory STM (Mazzoni et al., 1996). Further, correlates of auditory STM for tone frequencies have been reported in primary auditory cortex (Gottlieb et al., 1989; Sakurai, 1990, 1994) as well as auditory thalamus (Sakurai, 1990). Thus, like the visual system, auditory STM may rely on the coordinated action of multiple brain areas including the PFC, temporal and parietal sensory association areas, primary sensory cortex, and thalamus.

Despite the moderate amount of progress toward understanding the neural substrates of auditory STM, there are still many remaining questions. For example, very few studies have investigated non-spatial STM using purely auditory tasks that include complex, naturalistic sound types such as conspecific vocalizations, which may be important for communication (Poremba et al., 2013). Our lab has recently conducted neurophysiological recording studies in the PFC (Plakke et al., 2013) and dorsal temporal pole (dTP), the rostral-most portion of the superior temporal gyrus (Poremba et al., 2003, 2004; Poremba and Mishkin, 2007; Ng et al., 2014), in an effort to fill these gaps in knowledge. Non-human primate subjects were trained to perform a same/different DMS task, wherein sample and test sounds were separated by a 5-s retention interval. Subjects were trained to press a button (“go” response) if the sounds were identical, and to withhold button presses (“no-go” response) if the sounds were non-identical. In the interest of separating sound-evoked responses from activity related to the button press and/or rewards, subjects were required to wait 1 s after the test sound had terminated to make their response. During the retention interval, portions of cells in both PFC and dTP exhibited significant changes in firing rate, though in smaller proportion, and with less consistency than has been reported in most unit-recording studies of visual STM. In the PFC, matching test sounds often evoked enhanced firing rates relative to non-matching sounds as well as the sample (Plakke et al., 2013). On average, these firing rates remained elevated throughout the wait period before a behavioral response was made. In dTP on the other hand, matching sounds were typically associated with suppressed firing rates that were observed very early during the sound presentation period (Ng et al., 2014). Perhaps as a result of top–down feedback originating in PFC, firing rates on match trials later increased during the wait period such that they exceeded firing rates on non-match trials.

Taken together, the findings that matching sounds which require “go” responses produce elevated firing rates in PFC but initially suppress firing rates in dTP suggest that separate neural mechanisms may be involved in differentiating matching vs. non-matching sounds. One possibility is that early match suppression effects in dTP and match enhancement effects in PFC (and later in dTP) reflect bottom–up processes involved in detecting changes in the acoustic environment (e.g., Jääskeläinen et al., 2007), and top–down processes involved in detecting events that are needed to guide prospective behavior, respectively. If true, early match suppression and late enhancement effects similar to those observed in dTP might be observed at earlier levels of the auditory system, including primary auditory cortex (A1). On the other hand, cue enhancement and suppression effects and other task-driven modulations of neurophysiological activity, such as delay-related changes in firing rate, might not be observed at this early stage in the auditory processing stream. To investigate these possibilities, neurophysiological activity was recorded from A1 in subjects performing an auditory STM task.

## Methods

### Subjects and surgery

Two adult macaque monkeys (*Macaca mulatta*) served as subjects for this experiment (monkey A: female; monkey O: male). The subjects were the same as those used in prior experiments from our lab investigating neural correlates of auditory STM in PFC (Plakke et al., 2013) and dTP (Ng et al., 2014). Both animals had extensive prior experience with auditory STM tasks and passive sound exposures (Plakke et al., 2008, 2013; Ng et al., 2009, 2014; Bigelow and Poremba, 2013a). The monkeys were housed under a 12:12 light:dark cycle in individual cages with *ad libitum* access to water and controlled feeding schedules. Subjects were fed after training each day (Harlan monkey diet plus fruit, vegetables, and treats) and maintained above 85% of their free-feeding weight throughout the duration of the experiment. Prior to the experiment, the monkeys were surgically prepared with electrophysiological recording chambers. Subjects were sedated with ketamine (10 mg/kg) and anesthetized with isoflurane (1–2%). Prior to surgery, each monkey was scanned with magnetic resonance imaging (MRI: 2T Sigma unit; GE Medical Systems, WI) to locate the coordinates of A1 and to verify the placement of electrodes within the recording grid (see below). Using a stereotaxic apparatus (David Kopf Instruments, Tujunga, CA), an angled 45-degree recording chamber (Crist Instruments, Hagerstown, MD) was implanted on the skull over the left hemisphere, centered at −2 mm posterior and −23 mm lateral of stereotaxic 0,0 (Saleem and Logothetis, 2007), and its position was secured with titanium screws and dental acrylic. A stainless steel head post was attached to the back of the skull to enable head restraint during electrophysiological recordings. Antibiotics and analgesics were administered as needed following surgery. Recording chambers were routinely cleaned with antiseptics using sterile instruments to inhibit infection. All surgical and experimental procedures conformed to standards provided by the National Institutes of Health and were approved by the Institutional Animal Care and Use Committee at the University of Iowa.

### Apparatus and recording procedure

Experiments were conducted in a double-walled sound attenuation chamber (Industrial Acoustics Company, Bronx, NY). Subjects sat in a custom-made primate chair that allowed free arm movements while restraining head movements with a bar that attached to the head post. Sounds were presented through a central speaker located approximately 40 cm from the head region. Responses were made via a single acrylic button positioned 3 cm below the speaker. Small food rewards were dispensed from a pellet dispenser (Med Associates, Georgia, VT) into a dish located 3 cm below the response button. An overhead “house light” provided illumination for the duration of the experiment, and a second overhead light provided additional illumination during the intertrial interval (ITI). Custom-designed software (LabView, National Instruments, Dallas, TX) controlled and recorded all task events. A small overhead camera with microphone allowed audiovisual observation by the experimenter.

At the outset of each session, a multielectrode system was used to lower 1–4 tungsten microelectrodes (1–3 MΩ impedance; FHC Inc., Bowdoin, ME) into A1. Each electrode was held by a 23-g sterile guide cannula positioned in an x-y grid attached to a micromanipulator, and was advanced to the region of interest using a computer-controlled electrode drive system (NAN Instruments, Nazareth, Israel). Spiking activity was extracted by applying a band-pass filter (0.5–10 kHz) to the raw extracellular signal. The resulting spike waveforms were amplified, digitized, and displayed in real time using a Multichannel Acquisition Processor (Plexon Inc., Dallas, TX), with spike times saved to hard disk at 40 kHz. Task events such as stimulus presentations and behavioral responses were recorded concurrently with the neurophysiological data. Both single- (SUA) and multi-unit activity (MUA) were collected. At many recording sites, it was possible to isolate SUA using a combination of online (dual window discriminators; Sort Client, Plexon Inc., TX, USA) and offline (e.g., principal components analysis, template matching; Offline Sorter, Plexon Inc., Dallas, TX) spike-sorting techniques. MUA was defined as the unsorted spike activity exceeding a site-specific amplitude threshold. Neurophysiological recordings were initiated after one or more single units had been isolated. A total of 334 units (SUA: 160 units; MUA: 174 units) were recorded and analyzed.

The position of A1 was estimated using electrode coordinates based on the recording grid position and electrode depth in conjunction with the animals' MRIs. The locations of all units included in the analyses below were estimated between −22 and −28 mm from bregma in the medial to lateral plane, and between 9.5 and 4 mm in the anterior to posterior plane (Saleem and Logothetis, 2007), covering the full area of A1, with multiple dorsal to ventral electrode penetrations. Following the STM task, each unit was passively exposed to a range of 56 pure tones and band-passed noise stimuli (500-ms duration) with center frequencies spanning 0.1–18.9 kHz. Each stimulus was repeated 9–11 times in pseudorandom order separated by a variable interstimulus interval (mean: 1320 ms; range: 1200–1500 ms). Consistent with the estimated recording location of the electrodes, most of the available units (300/313) exhibited significant frequency selectivity (21 of the units were lost before the passive exposure phase and so were not analyzed), where the firing rate elicited by the best tone frequency exceeded the mean response elicited by the remaining frequencies by at least two standard deviations (example units shown in Figure S1). In addition, mean peak amplitudes and latencies elicited by tones and noise were assessed for each unit. Previous studies have indicated that belt areas exhibit significantly greater peak amplitudes with shorter latencies in response to noise stimuli compared to tones, whereas no significant differences are observed in A1 (e.g., Lakatos et al., 2005; Kayser et al., 2008). Using repeated-measures analysis of variance (ANOVA), no significant differences in peak amplitude [*F*_(1, 312)_ = 1.1, *p* > 0.05] or latency [*F*_(1, 312)_ = 2.1, *p* > 0.05] elicited by tones and noise were detected at the unit population level. Thus, in conjunction with the estimated anatomical coordinates, the physiological results suggest that our unit population was recorded primarily within A1, although it is possible that some units were recorded from the immediately adjacent cortical fields.

### Short-term memory task

**Figure 1:**
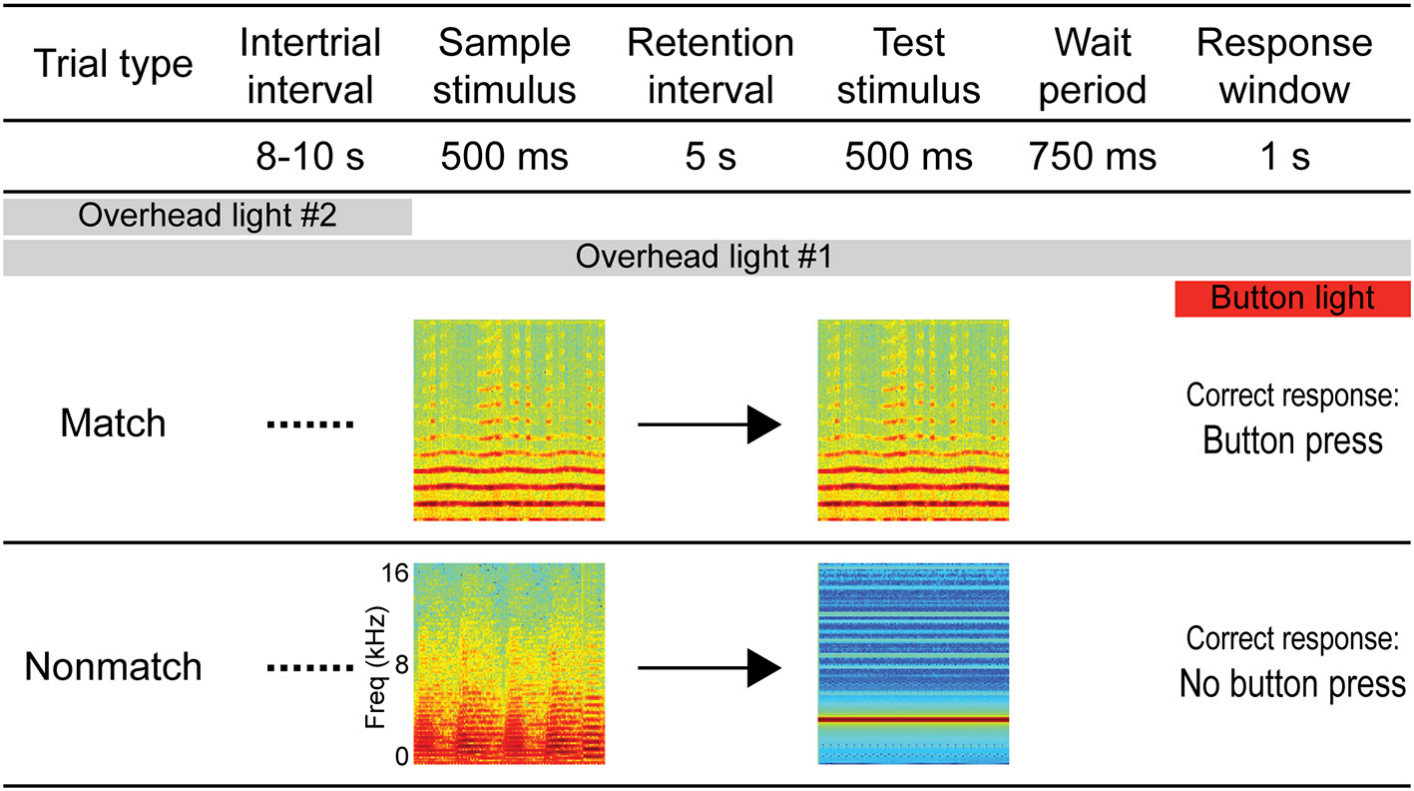
**Diagram of the auditory short-term memory task.** Each trial consisted of 500-ms sample and test sounds separated by a 5-s retention interval. For match trials, the sounds were identical and the correct response was a button press, whereas for non-match trials the sounds were non-identical and the correct response was to withhold from pressing the button. Sample and test sounds were pseudorandomly selected for each trial from a variety of naturalistic and artificial sound exemplars (see Methods). A pre-response wait period followed the test stimulus, after which the response button was illuminated to signal the response window. Responses outside of the response window (e.g., during the sound presentations or wait period) aborted the trial and these trials were not included in subsequent analyses. Overhead lighting provided constant low-level illumination throughout the session, and a second overhead light was turned on during the ITI to serve as a cue by which trials could be segregated.

The auditory STM task employed in this experiment was the same/different variation of the DMS task (D'Amato and Worsham, 1974; Wright, 2007), which is suitable for auditory stimuli. A schematic diagram of the task is depicted in Figure 1. Following a variable ITI (mean: 9 s, range: 8–10 s), each trial began with a sample stimulus, followed by a 5-s retention interval, after which a test stimulus was presented. Similar to previous experiments from our lab (Plakke et al., 2013; Ng et al., 2014), a 750-ms pre-response wait period began after the test stimulus had terminated. This was included to ensure that sound-evoked responses were not contaminated by artifact related to behavioral responses or reward expectancy (e.g., Brosch et al., 2005; Yin et al., 2008). Following the pre-response wait period, the response button was illuminated by an orange backlight for 1 s, indicating the response window. Responses outside of the response window (e.g., during the sound presentations or wait period) aborted the trial and these trials were not included in subsequent analyses. For trials on which the sample and test sounds were identical (*same* or *match* trials), the correct response was defined as a button press (“go” response). For trials on which the sample and test sounds were non-identical (*different* or *non-match* trials), the correct response was defined as the absence of a button press (“no-go” response). Responses were subject to an asymmetric reinforcement contingency in which correct “go” responses on match trials were rewarded with a small food pellet and incorrect button presses on non-match trials (“false match” responses) were occasionally punished by a brief, mild air puff presented indirectly from a distance of approximately 15 cm from the animal. During the monkeys' initial training, false match responses regularly resulted in punishment; however, following acquisition of the task, approximately 1/10 of the “false match” responses were punished on a variable schedule. Similar DMS tasks using the go/no-go paradigm and asymmetric reinforcement contingency have been used in previous studies of auditory STM in monkeys and other animals as they facilitate learning the same/different rule (Stepien and Cordeau, 1960; Nelson and Wasserman, 1978; Kojima, 1985; Colombo and D'Amato, 1986; Ng et al., 2009; Munoz-Lopez et al., 2010). Each session comprised 200 trials with an equal number of match and non-match trials presented in pseudorandom order.

### Stimuli

One of 12 stimulus sets was pseudorandomly selected for each experimental session which contained one exemplar of each of the following eight sound types: conspecific monkey vocalizations, human vocalizations, animal vocalizations, natural/environmental sounds, music samples, synthetic sounds, pure tones, and band-passed noise. All stimuli were trimmed to 500 ms with the exception of several vocalization stimuli that were shorter than 500 ms. The sounds were volume normalized using Audition (Adobe Systems, San Jose, CA) and presented at 72 ± 5 dB. Spectrograms and temporal envelopes for each of these stimuli are shown in Figure S2. Monkey vocalizations were recorded at a natural monkey reserve in South Carolina, USA (by Amy Poremba), and included coos, grunts, screams, and shrill barks. Human vocalizations included various speech and non-speech vocal sounds from a variety of male and female speakers. Animal vocalization exemplars were drawn from a variety of birds and non-primate mammals. Natural and environmental sounds included recordings of events such as flowing water and rushing wind. Music samples were recordings of instrumental music, e.g., a three-note sequence played on a piano. Synthetic sounds comprised sounds that do not occur naturally, e.g., they were generated electronically with a synthesizer. Pure tones and band-passed noise exemplars were digitally generated with a range of center frequencies spanning 1083–8820 Hz. Each sound was presented with equal frequency as the sample and test sounds on both match and non-match trials.

In addition to these eight sound types, two variations of a white noise burst were included in each session and presented on the same number of trials as the other sounds. For match trials, the white noise burst comprised two 200-ms periods of noise separated by a 100-ms silence gap. For non-match trials, the noise burst comprised four 100-ms periods of noise separated by three silence gaps (100 ms total). These stimuli were included, among other objectives, to investigate whether subjects were sensitive to the differential contingencies (match vs. non-match) associated with the subtle temporal variations in the noise bursts. We found only limited evidence that they did so: accuracy did not change when the white noise variation associated with match trials was presented, and accuracy benefitted only modestly when the white noise variation associated with non-match trials was presented as the test sound, but not as the sample. In light of recent findings by Scott et al. (2013), the failure of our subjects to exploit the information contained in the noise burst stimuli is not surprising. Scott and colleagues found that monkeys performing an auditory DMS task made little use of the temporal information contained in a variety of natural and artificial sounds, similar to those used in our experiment. Instead, the monkeys relied heavily upon spectral content of the sounds in making the match/non-match decision. Thus, non-matching sound pairs with uncorrelated spectra and disparate harmonics-to-noise ratios (HNRs) were associated with higher non-match decision rates. Indeed, the white noise bursts in our study were spectrally distinct from the remaining sound types, which may have contributed to the modest benefit in accuracy when presented as a non-matching test stimulus. Thus, for the purposes of the current study, the noise burst stimuli were not evaluated separately from the other sound types.

### Data analysis

Behavioral data were analyzed by computing mean accuracy, response latency, and d-prime values for each session. Comparisons between trial types were tested using ANOVA with the session means as individual data points. Subjects occasionally quit participating in the task prior to the end of the programmed session. These trials were not included in behavioral or neurophysiological analyses to ensure that any observed effects were attributable to mnemonic factors, rather than motivation or arousal. As in previous studies (Bigelow and Poremba, 2013a,b), for sessions in which subjects made no responses during the last 20 trials or more, the final response was considered as the end of the session (6.0% of total trials).

The sorted SUA and MUA data were exported to neurophysiological data analysis software (NeuroExplorer, Nex Technologies, Littleton, MA), wherein spike activity related to task events such as the sample and test sounds was evaluated using peristimulus time histograms (PSTH). Unless otherwise indicated, average firing rates were sampled in 20-ms bins. For individual unit analyses, single trial means comprised individual data points (note that non-identical numbers of trials were typically used for comparisons between conditions, such as match vs. non-match, and were therefore considered independent). For population analyses, the session means for each unit (collapsed across individual trials) served as individual data points. Population analyses combined SUA and MUA except where noted (cf. Kayser et al., 2008). Changes in firing rate during the retention interval were assessed with ANOVA plus *post-hoc* tests comparing 10 successive 500-ms segments of the retention interval to a 500-ms pretrial baseline period (*p* < 0.05, Fisher's LSD). Differences in firing rate between conditions (e.g., match vs. non-match) were tested with ANOVA using a 100-ms sliding window, advancing in 20-ms increments (cf. Apicella et al., 1997; Darbaky et al., 2005; Chandrasekaran and Ghazanfar, 2009). Effects were only considered significant in cases where significant differences (*p* < 0.05) were obtained for two or more consecutive steps. Because population analyses included a relatively large number of units, and since additional comparisons were made between conditions during the sample stimulus resulting in a larger number of tests, a more conservative alpha level was adopted (*p* < 0.005).

## Results

### Behavioral results

The subjects attained, on average, 65.5% overall accuracy based on 75 total behavioral sessions. This modest level of accuracy is common for non-human primates performing auditory STM tasks, even after extensive training (Fritz et al., 2005; Scott et al., 2012, 2013; Bigelow and Poremba, 2013a, 2014). Although relatively poor compared to studies of visual STM in monkeys (e.g., Fritz et al., 2005), a comparison of the number of correct and incorrect trials per session confirmed that performance was well above chance [*F*_(1, 74)_ = 315.1, *p* < 0.05). As in prior studies from our lab using the same subjects as well as other animals (Bigelow and Poremba, 2013a,b; Plakke et al., 2013; Ng et al., 2014), a strong “go” bias was observed: subjects correctly responded on 75.5% match trials (“hits”), but incorrectly responded on 44.1% of non-match trials (“false alarms”; mean d-prime: 1.1). Also consistent with our previous experiments was the finding that correct hits were made significantly faster (response latency = 394 ms) than false alarms (response latency = 462 ms; *F*_(1, 74)_ = 397.6, *p* < 0.05).

### Retention interval

**Figure 2:**
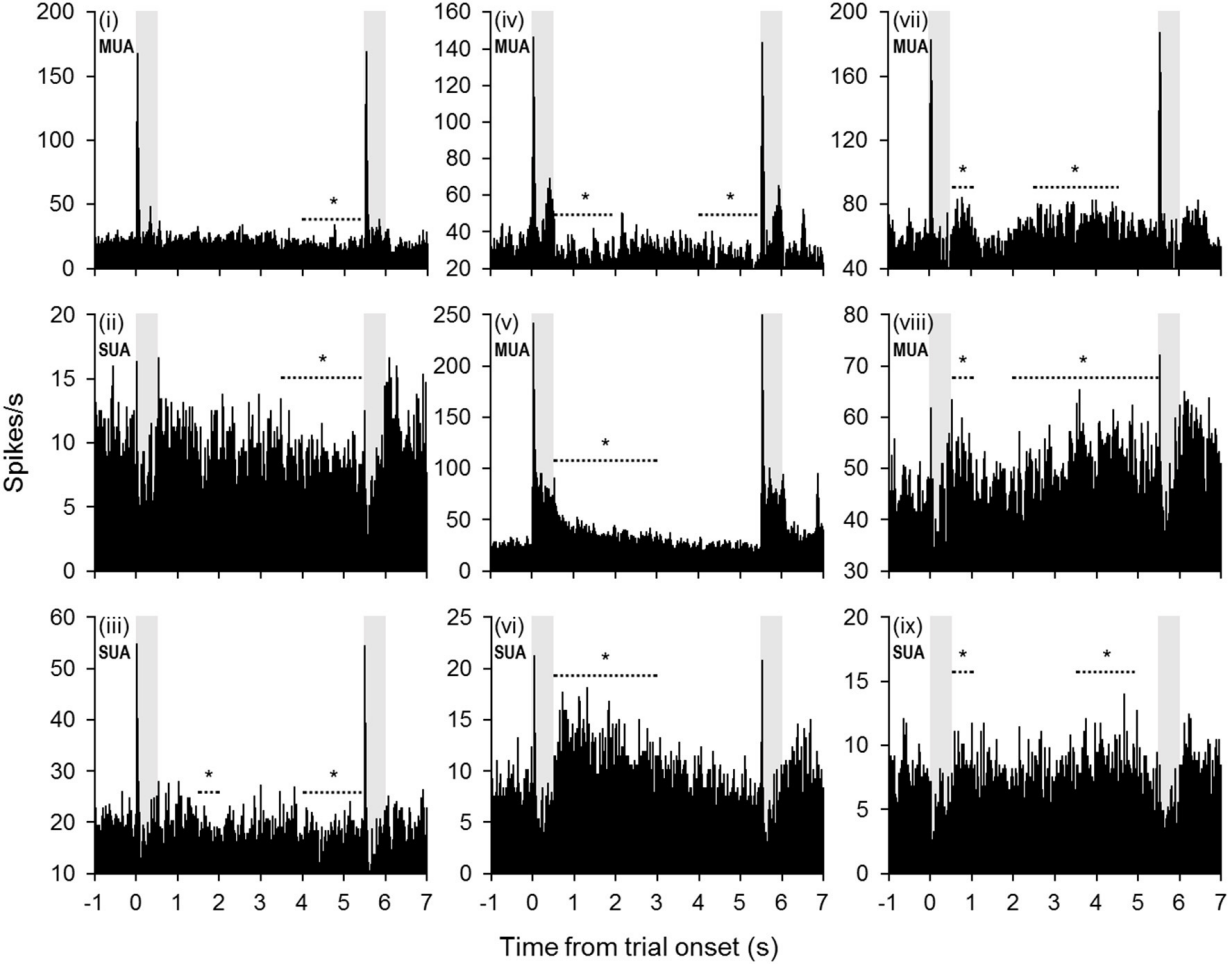
**Example units with significant changes in firing rate during the retention interval.** Many units exhibited a diminishing firing rate throughout the retention interval (units i–vi). In some cases (units i–iv), the firing rate fell below baseline during the latter portion of the retention interval. In other cases (units v–vi), the firing rate returned to baseline levels from a significantly elevated firing rate earlier in the retention interval. Other units exhibited a trend toward increased firing rates during the retention interval (units vii–ix). The periods denoted by dashed lines with asterisks indicate successive 500-ms bins that were significantly different from baseline (mean firing rate 500 ms prior to trial onset). Shaded gray areas indicate sample and test stimulus presentation periods.

**Figure 3:**
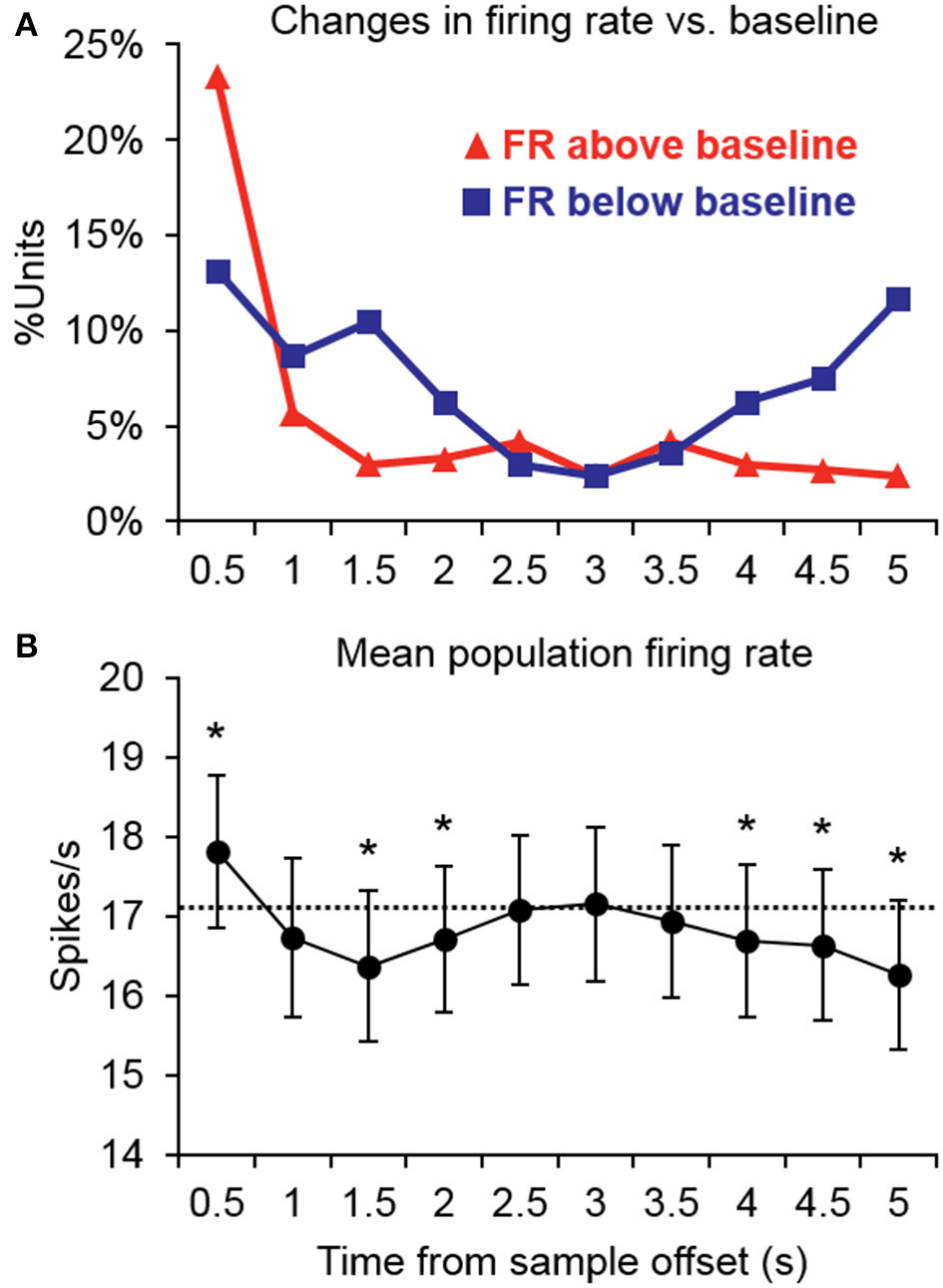
**Summary of significant changes in firing rate from baseline during the retention interval. (A)** Many units exhibited increased firing rates immediately after the offset of the sample stimulus, but for the majority of units, firing rates returned to baseline thereafter. Suppression was somewhat less common immediately following sample stimulus offset, but was observed more frequently further into the retention interval. Suppression was also more common in the latter retention interval bins prior to test stimulus onset. FR, firing rate. **(B)** Consistent with the firing rate changes observed in individual units, the mean population firing rate was briefly elevated at the offset of the sample stimulus, but then temporarily fell below baseline. After returning to baseline near the midpoint of the retention interval, firing rates again fell significantly below baseline during the latter portion of the retention interval prior to the test stimulus. Asterisks indicate retention interval periods that differed significantly from baseline (500 ms prior to trial onset) indicated by the dashed line.

Changes in firing rate during the retention interval were assessed by comparing the mean firing rate during the pretrial baseline (500 ms prior to sample onset) to 10 successive 500-ms segments during the retention interval. Example units exhibiting significant changes from baseline in one or more segments of the retention interval are shown in Figure 2, and a summary of units with significant changes from baseline during each segment is presented in Figure 3A. The largest portion of units (23.4%) exhibited an increased firing rate relative to baseline in the first 500-ms period of the retention interval (i.e., the sample offset period). However, for the majority of these units, the elevated firing rate did not persist into the retention interval any further. Although suppressed firing rates relative to baseline were less common during the first segment of the retention interval, they were observed more frequently further into the retention interval (e.g., 2000 ms after sample offset). Also, more units exhibited suppressed firing rates (117 units; 35.0%) compared to elevated firing rates (93 units; 27.8%) for at least one 500-ms segment of the retention interval, with a large portion of suppression effects observed during the latter portion of the retention interval. Consistent with these observations, repeated ANOVA revealed that mean population firing rates varied significantly from baseline during the retention interval [*F*_(10, 3330)_ = 19.5, *p* < 0.05). *Post-hoc* tests (*p* < 0.05, Bonferroni correction) indicated that firing rates were briefly elevated at sample stimulus offset, but then became suppressed. After returning to baseline near the midpoint of the retention interval, firing rates again fell significantly below baseline during the last 1500 ms prior to test stimulus onset (Figure 3B).

The pattern of firing rate changes observed during the retention interval in the current study are similar in many ways to the results of previous studies from our lab investigating neuronal activity during auditory STM in the PFC (Plakke et al., 2013) and in dTP (Ng et al., 2014). Units in all three cortical areas exhibited significant increases or decreases in firing rate relative to baseline. In the current study, units more frequently exhibited reduced firing rates with the exception of the first bin of the retention interval (i.e., the sample offset period). In particular, suppressed responses were dominant during the latter portion of the retention interval, where firing rates were significantly below the pretrial baseline at the population level (Figure 3). Similar changes from baseline firing rates were not observed at the population level in either PFC or dTP (Plakke et al., 2013; Ng et al., 2014), suggesting a greater degree of suppression in A1.

In general, the percentages of units exhibiting changes from baseline during the retention interval in each of our studies (PFC, dTP, and A1) have been smaller than what has typically been reported in studies of visual STM in various cortical areas (Fuster and Alexander, 1971; Fuster and Jervey, 1981; Miller et al., 1996; Shafi et al., 2007). Moreover, in contrast to the sustained changes in firing rate in these studies, the units recorded in our experiments typically exhibited changes in firing rate that were transient or intermittent. Indeed, only 16 units (4.8%) in the present study exhibited significant changes from baseline for half of the retention interval or more, and only 1 unit exhibited sustained suppression throughout the entire retention interval. These findings also differ from a previous study of neuronal activity in A1 during an auditory STM task, which showed changes in firing rate (both increased and decreased) that were sustained throughout the entire retention interval (Gottlieb et al., 1989). However, the retention interval in that study was only 1 s, leading to the possibility that the responses might have returned to baseline during an extended retention interval. Moreover, in contrast to the go/no-go paradigm used in the present study, Gottlieb et al. (1989) trained their subject to perform a two-alternative forced-choice DMS task in which reward was available on every trial (pending correct responses). The differences in task contingencies may have thus contributed to the differences in firing rate changes during the retention interval, inasmuch as response and reward anticipation has been shown to influence neuronal activity in A1 (Brosch et al., 2005, 2011; Yin et al., 2008) and other cortical areas (Curtis and D'Esposito, 2003).

### Cue-evoked responses: individual unit analyses

**Figure 4:**
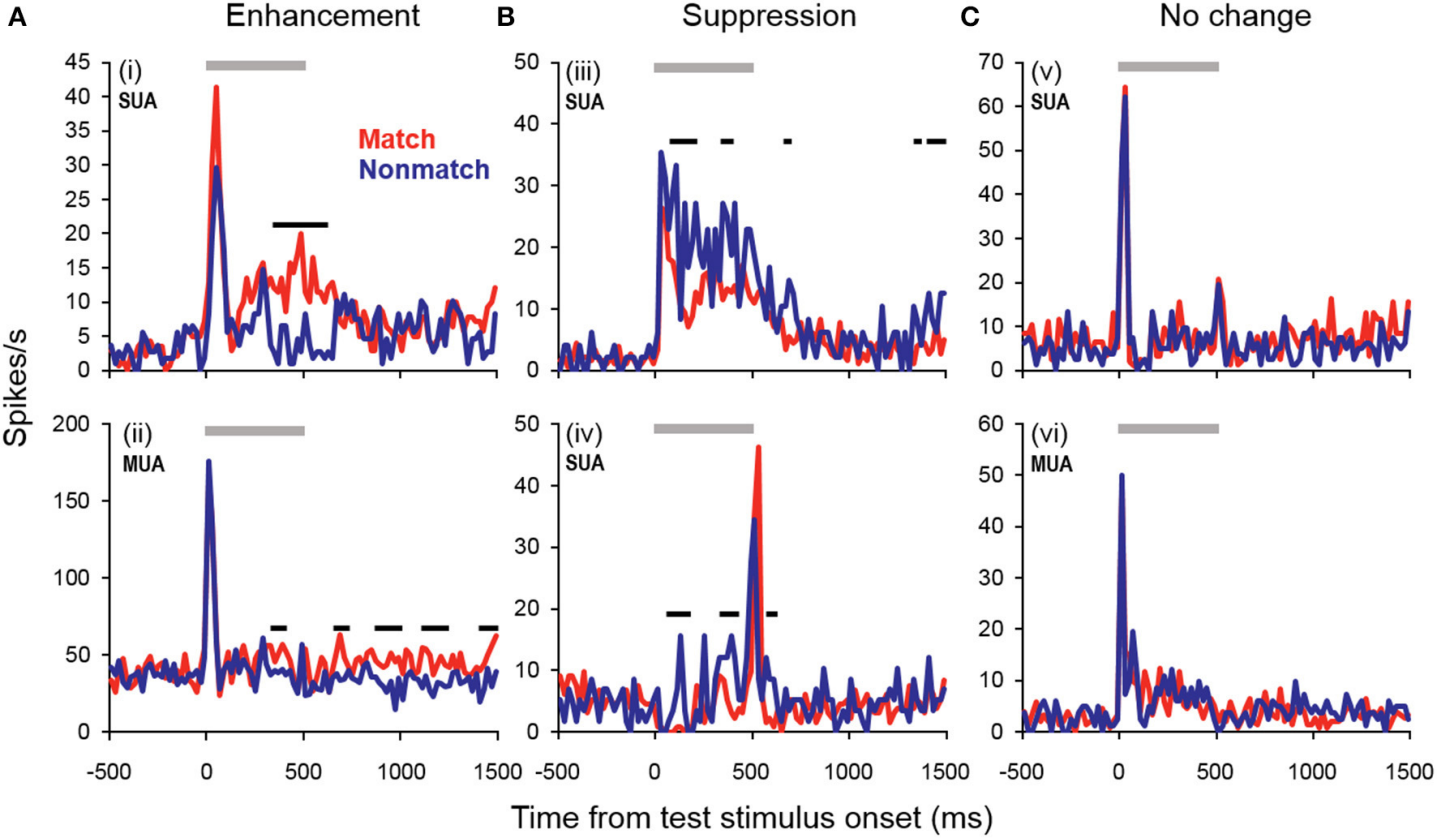
**Example units showing match enhancement, suppression, or no change. (A)** Example units for which enhanced firing rates were elicited by matching compared to non-match test sounds. **(B)** Example units for which suppressed firing rates were elicited by matching compared to non-match test sounds. **(C)** Examples of units for which there were no significant differences in firing rate elicited by matching and non-matching test stimuli at any point during the cue, offset, or pre-response wait periods. Gray bars indicate the test stimulus presentation period. Black bars above the firing rate histograms indicate significant differences in firing rate between trial types (assessed with a 100-ms sliding window, advancing in 20-ms steps). Note that very brief changes in firing rate were not reported (such as those observed for unit i at stimulus onset and unit iv at stimulus offset), inasmuch as differences were only accepted if significant effects were obtained for two or more consecutive steps.

Cue enhancement and suppression effects were examined on an individual unit basis and at the population level by comparing firing rates between cue types using a 100-ms sliding window (20-ms step). Previous studies have observed “match enhancement” both during and after the cue presentation period (Plakke et al., 2013; Ng et al., 2014). To capture these possible effects, comparisons were made to test for potential differences in firing rate on match and non-match trials during the cue presentation period (0–500 ms from cue onset) as well as the offset period (0–500 ms from cue offset) and the pre-response wait period (500–1000 ms from cue offset). Units that exhibited significant positive differences (*p* < 0.05) for two or more consecutive bins were considered to show enhancement effects, whereas units that exhibited significant negative differences for two or more consecutive bins were considered to show suppression. The results are summarized in Table 1, with individual unit examples presented in Figure 4. In general, a higher proportion of units exhibited match enhancement compared to suppression (Table 1). An exception to this general outcome was that, during the cue period only, the single-unit subpopulation more frequently exhibited suppressed responses to matching test stimuli. The proportion of units exhibiting significant match enhancement effects increased as the trial progressed from the cue presentation period to the cue offset and pre-response wait periods.

**Table 1:** **Match enhancement and suppression effects for individual units**.

	**Enhancement**			**Suppression**		
	**Cue (%)**	**Offset (%)**	**Wait (%)**	**Cue (%)**	**Offset (%)**	**Wait (%)**
MUA	28.2	35.6	43.1	17.8	16.1	14.4
SUA	18.8	21.9	25.6	23.1	18.8	14.4
Overall	23.7	29.0	34.7	20.4	17.4	14.4

*Enhancement and suppression effects based on comparison of mean firing rates between cue types using a 100-ms sliding window, advancing in 20-ms steps. Effects are reported where significant differences were obtained for two or more consecutive bins. “Cue” = cue presentation period; “Offset” = 500 ms post-cue period; “Wait” = 500 ms post-offset period. Percentages based on 334 units (SUA: 160 units; MUA: 174 units)*.

### Cue-evoked responses: population analyses

**Figure 5:**
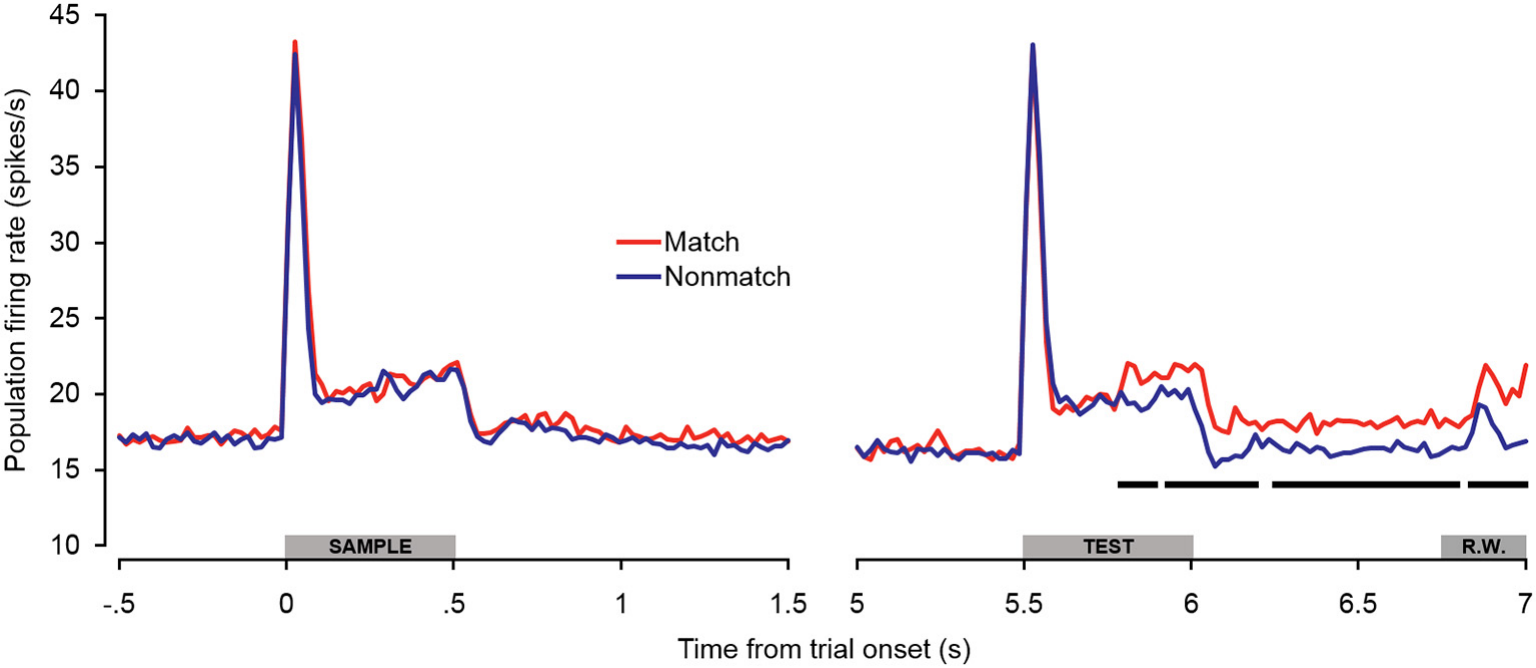
**Population spiking activity during auditory short-term memory task.** Firing rates elicited by matching and non-matching test stimuli are depicted in the right panel, and firing rates elicited by the sample stimuli are shown in the left panel as a control comparison. Beginning during the latter portion of the test stimulus presentation period, firing rates became significantly higher for match compared to non-match trials. This difference was sustained with minimal interruption throughout the offset and pre-response wait periods. The black bars below the firing rate histograms indicate significant differences between trial types (assessed with a 100-ms sliding window, advancing in 20-ms steps). Differences were only accepted if significant effects were obtained for two or more consecutive steps. The gray bars above the abscissae indicate the sample and test stimulus presentation periods (0–500 ms from cue onset) as a well as the onset of the response window (R.W.).

In general, the trends observed in the individual unit enhancement and suppression analyses were reflected in the population-averaged firing rate shown in Figure 5. There were no significant differences between trial types during the sample stimulus period or retention interval, or during the peak response evoked at the onset of the test stimulus (~0–100 ms post-stimulus onset). However, significantly enhanced firing rates were observed beginning approximately 300 ms after test onset and continuing throughout the offset and pre-response periods. At this latency, the significant match enhancement effects observed in the present study follow those observed in the PFC by at least 100 ms (Plakke et al., 2013). Moreover, the magnitude of the match enhancement effects in A1 was relatively modest compared to those reported in PFC. These observations are consistent with previous studies suggesting task-specific response modulation in A1 likely reflects feedback from other cortical areas including PFC, where task-relevant information is identified and responses are selected (Scheich et al., 2007). In dTP, firing rates elicited by matching test sounds were significantly elevated over non-matching test sounds only during the late offset and pre-response wait periods (Ng et al., 2014). This suggests that task-related feedback originating in higher cortical areas such as PFC may reach A1 first, and in turn propagate along the superior temporal gyrus.

**Figure 6:**
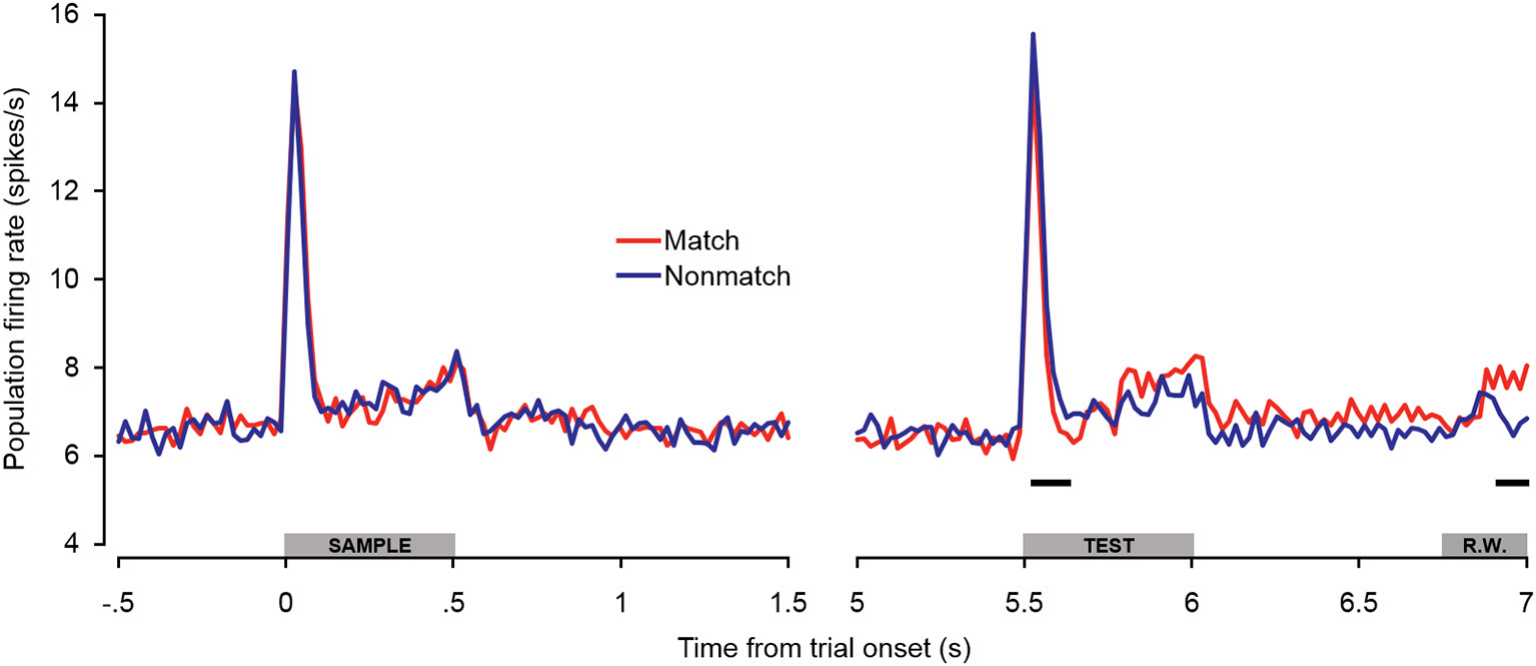
**Single-unit subpopulation spiking activity during auditory short-term memory task.** Firing rates elicited by matching and non-matching test stimuli are depicted in the right panel, and firing rates elicited by the sample stimuli are shown in the left panel as a control comparison. Similar trends were observed in the population (Figure 5) and single-unit subpopulation analyses. However, early match suppression effects reached significance only in the single unit subpopulation. In addition, the elevated firing rates beginning during the latter portion of the test stimulus were less robust, reaching significance only during the late pre-response period. The black bars below the firing rate histograms indicate significant differences between trial types (assessed with a 100-ms sliding window, advancing in 20-ms steps). Differences were only accepted if significant effects were obtained for two or more consecutive steps. The gray bars above the abscissae indicate the sample and test stimulus presentation periods (0–500 ms from cue onset) as a well as the onset of the response window (R.W.).

Because there were differences in the percentages of single- and multi-units that exhibited match enhancement effects (Table 1), a subpopulation analysis was conducted that included only the single units (Figure 6). The general trends observed in the single-unit subpopulation were similar to those in the entire population analysis, although fewer differences between match and non-match trials reached statistical significance. One of the most substantial differences was that significant early match suppression effects were observed in the single-unit subpopulation. Because comparisons were made between 100-ms averages (advancing in 20-ms steps), these effects could have occurred as early as 40–60 ms post-stimulus onset—a latency comparable to the match suppression observed at 30–60 ms post-stimulus onset in dTP (Ng et al., 2014).

The observations of both early match suppression and late match enhancement effects in both A1 and dTP lend support to the possibility of separate neural mechanisms enabling auditory STM and ultimately differential behavioral responses on match and non-match trials. Although the mechanisms underlying reduced firing rates for repeated sounds are still under debate (Ng et al., 2014), early match suppression could reflect bottom–up stimulus-specific adaptation effects produced by local recurrent connections and input from thalamus and other cortical areas (Jääskeläinen et al., 2007; Liu et al., 2009; Ng et al., 2014). Indeed, modest adaptation effects have been observed in passive-exposure paradigms at interstimulus intervals of up to 5 s (Werner-Reiss et al., 2006). On the other hand, the ensuing elevated firing rates observed for matching sounds might reflect top–down feedback from higher cortical areas such as PFC, which are predominantly involved in integrating task-relevant sensory information and response selection.

One final observation that was evident in the population average firing rate (Figure 5) was a small excitatory response beginning approximately 120 ms into the response window. This response was apparently elicited by the orange backlight of the response button that signaled the response window. These modest light-evoked responses are consistent with previous studies demonstrating activation of A1 by non-acoustic stimulation including visual, somatosensory, and motor events, particularly if they are related to an auditory task in trained subjects (Fu et al., 2003; Brosch et al., 2005; Ghazanfar et al., 2005; Scheich et al., 2007, 2011; Kayser et al., 2008; Yin et al., 2008).

### Error trials

**Figure 7:**
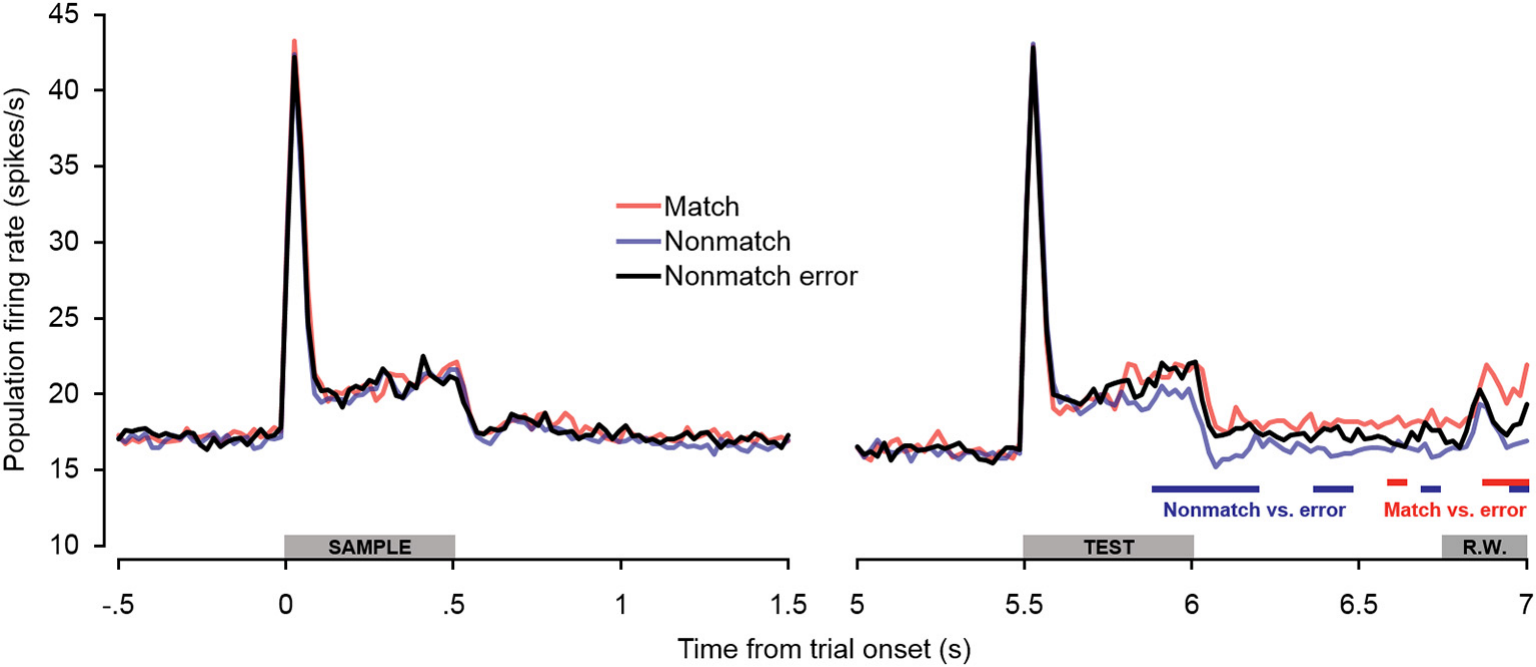
**Population spiking activity observed on error trials during auditory short-term memory task.** Firing rates elicited by sample and test stimuli are depicted in the left and right panels, respectively. No significant differences in firing rate were observed for non-match error trials during pretrial baseline, the sample stimulus presentation period, or the retention interval. During the latter portion of the test stimulus period, firing rates on non-match error trials were significantly higher than on correct non-match trials (similar to match trials). During the cue offset and pre-response wait periods, firing rates on non-match error trials were intermediate between correct match and correct non-match trials. The blue and red bars below the firing rate histograms indicate significant differences between non-match error trials and correct non-match and match trials, respectively, (assessed with a 100-ms sliding window, advancing in 20-ms steps). Differences were only accepted if significant effects were obtained for two or more consecutive steps. The gray bars above the abscissae indicate the sample and test stimulus presentation periods (0–500 ms from cue onset) as a well as the onset of the response window (R.W.).

Additional analyses were conducted to test for potential differences in firing rates on non-match trials in which subjects incorrectly made button presses (false alarms). As seen in Figure 7, there were no differences in firing rate between non-match error trials and correct trial types during or immediately following the sample stimulus presentation period. Non-match error trials also did not differ from correct trials during the baseline firing rate or retention interval. During the latter portion of the test stimulus, however, firing rates on non-match error trials exceeded those observed on correct non-match trials, similar to what was observed on correct match trials. The differences were initially as great as those observed between correct match and non-match trials, but diminished later in the offset and pre-response periods, such that firing rates on non-match error trials eventually fell significantly below firing rates on correct match trials. The observation that firing rates on non-match error trials exhibit a relatively late increase in firing rate similar to correct match trials reinforces the idea that “match enhancement” may be related to top–down feedback reflecting response selection and/or anticipation, inasmuch as button presses were made for both trial types. This notion is corroborated by the observation that firing rates were similarly elevated on non-match error trials in PFC (Plakke et al., 2013). However, along with the differential response latencies observed for these two response types, the finding that elevated non-match error firing rates were not sustained to the same degree as firing rates on correct match trials suggests that processes underlying these two “go” trial types are not identical. Rather, neuronal and behavioral activity observed during non-match error trials appears to be intermediate between true match responses and correct non-match rejections, perhaps as others have suggested, reflecting reduced certainty in the behavioral choice (Benjamin and Bjork, 1996).

## Discussion

The foregoing results reveal that neurophysiological activity in A1 was associated with several aspects of auditory STM processing at the individual-unit and population levels. As in PFC and dTP, a modest number of units exhibited significant increases and decreases in firing rate during the retention interval. Moreover, stimulus-evoked responses were frequently modulated depending on the context in which the sounds were presented. Specifically, many units exhibited enhanced or suppressed responses depending on whether the sound was presented as a matching or non-matching test stimulus (Table 1, Figures 4–6). Analyses of error trials suggested these modulation effects in part reflected the subjects' perceptual decisions (Figure 7). Overall, these observations highlight flexible task-engagement of neurons at this early stage of auditory cortical processing.

As in our earlier studies of PFC and dTP (Plakke et al., 2013; Ng et al., 2014), both increases and decreases in firing rate were observed during the retention interval (Figures 2, 3). In the present study, increased firing rates were more frequently observed immediately following the sample stimulus, but the majority of significant effects thereafter reflected decreases in firing rate relative to baseline. In contrast to the results from PFC and dTP, these effects were sufficiently prevalent that firing rates fell significantly below baseline during the latter portion of the retention interval at the population level (Figure 3B). In studies of visual STM, sustained changes in firing rate during the retention interval have typically been interpreted as a correlate of mnemonic retention of a sensory cue for the guidance of prospective behavior (e.g., Shafi et al., 2007). Since these effects have been observed in many cortical areas, and have been shown to depend on interactions among these areas (e.g., Fuster et al., 1985), they are generally assumed to reflect sustained interactions among a distributed cortical/subcortical network that collectively enables neural representation of the sensory cue once it has passed from the environment. Delay-related changes in firing rate observed in the current study could reflect similar processes. Alternatively, since these firing rate changes were not sustained, but were mostly observed for 1 or 2 s following the sample stimulus and prior to the test stimulus, they could reflect mechanisms encoding the timing of the trial sequence (e.g., decreased firing rates near the end of the retention interval could reflect anticipation of the test stimulus). One additional possibility is that the suppression effects observed prior to the onset of the test stimulus could serve to increase the signal-to-noise ratio of the behaviorally-relevant sounds. Each of these possibilities deserves further experimental attention in studies using appropriate variations in task contingencies (e.g., variable vs. fixed retention interval).

In all three areas (PFC, dTP, A1), significant changes from baseline firing rates were generally not sustained, and were observed in a smaller proportion of units than typically reported in studies of visual STM (Fuster and Alexander, 1971; Fuster and Jervey, 1981; Miller et al., 1996; Shafi et al., 2007). One factor that might have contributed to these differences is the fact that, under the asymmetric response/reward contingency employed in our studies, subjects could not predict behavioral responses or rewards during the retention interval, which has been shown to modulate firing rates in PFC and other cortical areas (e.g., Kobayashi et al., 2002; Curtis and D'Esposito, 2003; Brosch et al., 2005, 2011; Shafi et al., 2007; Yin et al., 2008). Besides this difference in task contingency, each of our studies has used exclusively auditory stimuli as memoranda. Several earlier experiments investigating task-related activation of PFC neurons by auditory or visual stimuli during various STM and discrimination tasks invariably reported that fewer cells were activated by auditory stimuli, and that behavioral accuracy was lower for auditory trials (Watanabe, 1992; Kikuchi-Yorioka and Sawaguchi, 2000; Artchakov et al., 2007). These observations raise the possibility that delay-related changes in firing rate in PFC might be less robust for auditory stimuli, which could have downstream effects in A1 and dTP.

The early suppressed firing rates elicited by matching test stimuli relative to non-matching stimuli in the single unit subpopulation (Figure 6) are comparable to those observed in dTP (Ng et al., 2014). Although the mechanisms underlying match suppression are still under investigation (Grill-Spector et al., 2006), they may include local interactions among recurrent connections as well as inputs from thalamus and other cortical areas (Liu et al., 2009; Ng et al., 2014). Although it is possible that suppression effects could originate in A1 and subsequently bias firing rates in dTP, the early timing of the effects in both areas, and the fact that both areas receive direct input from auditory thalamus (Markowitsch et al., 1985), raise the possibility that they may arise independently in each area. In either case, these suppression effects appear to be the earliest indicator of a matching test stimulus. This signal could feed forward to higher cortical areas such as PFC, ultimately setting the stage for the distinct behavioral responses required by the STM task following repeated vs. different sound presentations.

**Figure 8:**
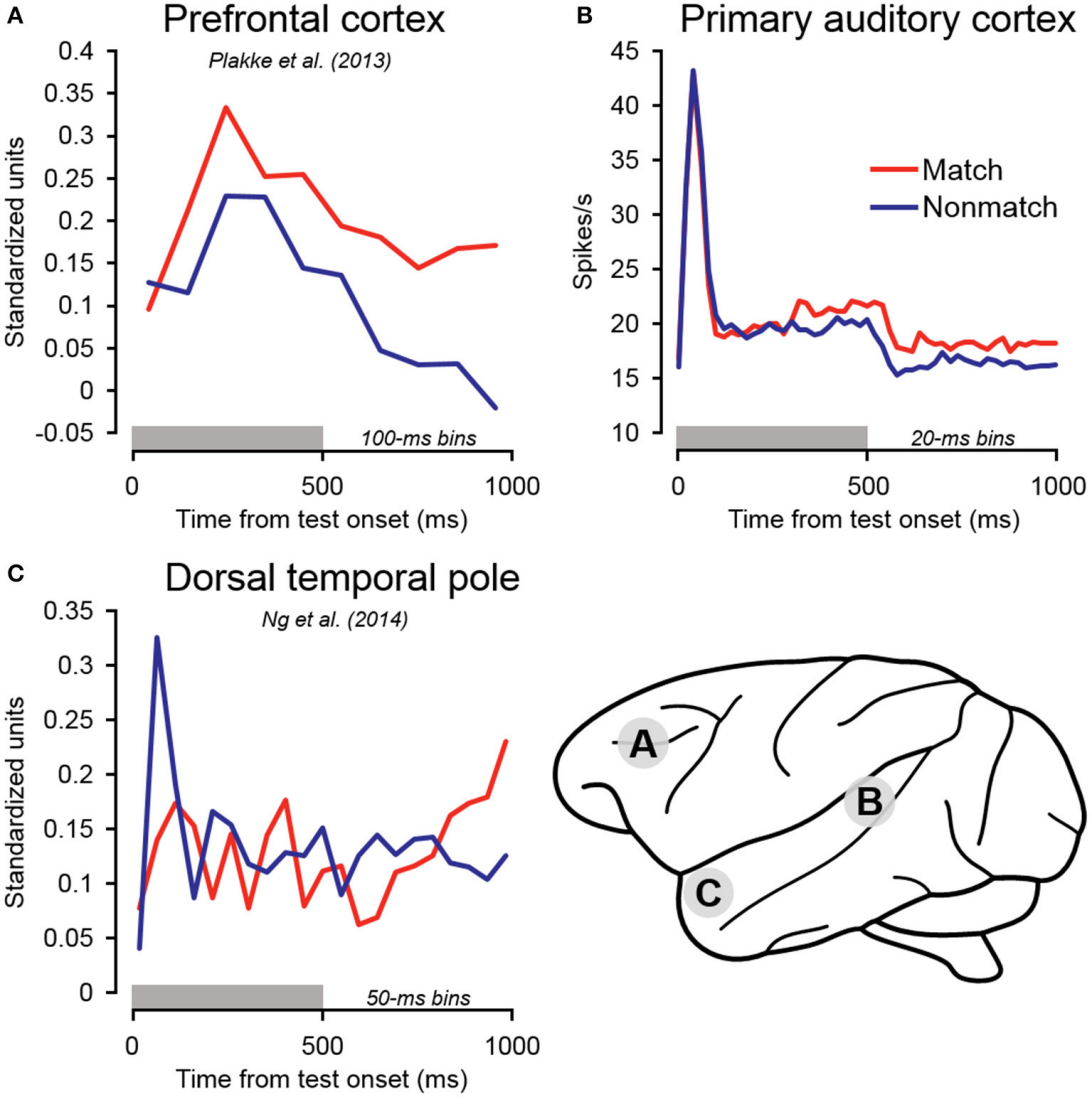
**Summary of population-averaged neurophysiological activity in three cortical areas for matching and non-matching test sounds. (A)** In PFC, firing rates on match trials became elevated relative to non-match trials during the early sound presentation period. Elevated firing rates were similarly observed on match trials in **(B)** primary auditory cortex and **(C)** dorsal temporal pole. However, these effects occurred later than in PFC, during the sound period or cue offset period, consistent with the notion that elevated firing in these areas may reflect top–down feedback originating in PFC. In contrast to these late match enhancement effects, significant suppression was observed on match trials in the early sound period in dorsal temporal pole. Early match suppression was also observed in auditory cortex in a portion of the individual units as well as in the single-unit subpopulation (Table 1, Figures 4, 6). The gray bars above the abscissae indicate the test stimulus presentation period (0–500 ms from cue onset). **(A)** Adapted from Plakke et al. (2013); **(C)** adapted from Ng et al. (2014). Each of the summarized experiments were conducted using the same subjects and auditory short-term memory task (see Methods for details).

Following the early suppression effects on match trials, firing rates became relatively elevated compared to non-match trials beginning approximately 300 ms after test stimulus onset, and remained elevated throughout the cue offset and pre-response periods (Figure 5). In dTP, firing rates on match trials similarly became relatively enhanced (Ng et al., 2014), but only beginning in the latter cue offset period—several 100 milliseconds later than the effects observed in A1. Of particular significance, match enhancement effects were also observed in PFC, and they were of larger magnitude and occurred earlier than both A1 and dTP (Figure 8; Plakke et al., 2013). These observations suggest that the relatively late elevated firing rates observed on match trials in A1 and dTP might be produced by top–down feedback originating in PFC, where task-relevant information is extracted. On the other hand, the relatively early match suppression effects might reflect bottom–up influences involved in detecting change in the acoustic environment. Together, these influences could work together to enable detection of matching sounds and selection of appropriate behavioral responses.

An additional observation supporting the hypothesis that match enhancement reflects top–down feedback reflecting behavioral choice is that firing rates were similarly elevated on non-match error trials, wherein subjects incorrectly reported a “match” decision. Similar elevated firing rates were observed on non-match error trials in the PFC (Plakke et al., 2013), as well as in dTP during the pre-response wait period (Ng et al., 2014). The late elevated firing rates on correct match trials and non-match error trials are therefore associated with the subjects' perceptual choices, rather than the actual same/different relationship of the sample and test sounds. Passive response modulation influences such as stimulus-order facilitation (e.g., Kilgard and Merzenich, 2002) are unlikely to account for these effects, inasmuch as the enhanced responses were observed on trials with both repeated (match) and distinct sounds (non-match error). Our observation of such effects in A1 corroborates earlier reports that A1 activity was correlated with subjects' perceptual decisions during auditory discrimination tasks (Sutter and Shamma, 2010; Niwa et al., 2012, 2013; Bizley et al., 2013).

The foregoing results can be added to a growing body of evidence that undermines the notion of A1 as a strictly unisensory area exclusively involved in processing acoustic information, e.g., detecting specific sound frequencies (Scheich et al., 2007; Weinberger, 2010). In addition to the correlates of perceptual choices discussed above, A1 activity has been shown to be modulated by non-auditory influences including visual and somatosensory events (Brosch et al., 2005; Ghazanfar et al., 2005; Bizley et al., 2007; Kayser et al., 2008; Scheich et al., 2011), motor activity (Brosch et al., 2005; Yin et al., 2008; Scheich et al., 2011), and reward feedback (Brosch et al., 2011). Scheich et al. (2007) have argued that these responses are unlikely to be generated by A1 itself, but rather reflect dynamic interactions with numerous other cortical areas that are driven by task demands. Our results are quite consistent with this view, since, with the exception of early match suppression, changes in firing rate associated with subjects' subsequent behavioral choices followed similar effects observed in PFC.

The studies reporting that fewer PFC neurons were activated by auditory stimuli during STM and discrimination tasks (Watanabe, 1992; Kikuchi-Yorioka and Sawaguchi, 2000; Artchakov et al., 2007) provide evidence for an important difference in the neural processes underlying visual and auditory STM in primates (see also Muñoz et al., 2009). Another significant difference was demonstrated in a study by Fritz et al. (2005), which showed that lesions of the perirhinal and entorhinal cortices significantly impair visual but not auditory DMS task performance. Notably, preoperative performance was superior for the visual task, but postoperative performance was similar for visual and auditory tasks. These performance outcomes are consistent with anatomical studies showing substantial projections to the rhinal cortices from visual and somatosensory, but not auditory cortical areas (Brown and Aggleton, 2001; Munoz-Lopez et al., 2010). These differences notwithstanding, other studies have provided evidence for similarities between auditory and visual STM circuits, such as a prominent role of the PFC in identifying task-relevant events and selecting appropriate behavioral responses, the involvement of other cortical areas including primary sensory cortex, and similar physiological phenomena including match enhancement and delay-related changes in firing rate. Thus, the available evidence reveals both substantial similarities and differences in neural processes underlying visual and auditory STM.

The current results contribute to a small but growing body of literature casting light on the neural processes underlying auditory STM. In combination with our prior studies of PFC and dTP, the current study strengthens the idea that distinct neural mechanisms may be involved in mediating the match/non-match decision during the auditory DMS task. Specifically, early bottom–up processes might enable the basic distinction of repeated vs. non-repeated sounds, and top–down influences might reflect selection of the appropriate behavioral response. In addition, all three studies have revealed that changes in firing rate during the retention interval are generally less robust during auditory STM. Because these types of activity have been shown to be important for performance of visual STM tasks, the less robust auditory effects might be related to the inferior performance that has been observed in numerous studies of auditory STM in primates (Cohen et al., 2005; Fritz et al., 2005; Munoz-Lopez et al., 2010; Scott et al., 2012; Bigelow and Poremba, 2014). Notwithstanding the contributions of the current experiment and other recent studies, our understanding of the neural substrates of auditory STM remains largely incomplete. For example, simultaneous recordings from multiple cortical and subcortical areas, perhaps paired with lesions or inactivations, could be conducted to directly test the speculative possibility that early match suppression and late enhancement effects represent bottom–up influences and top–down influences from PFC and other cortical areas, respectively. Additional studies are also needed to clarify the extent to which auditory and visual STM depend on similar neural processes and circuitry. In particular, studies using comparable auditory and visual STM tasks and ideally the same subjects hold the potential to explain differences observed at the behavioral level and aid in interpreting the function of neurophysiological phenomena such as cue-modulation effects and changes in firing rate during mnemonic retention.

### Conflict of interest statement

The authors declare that the research was conducted in the absence of any commercial or financial relationships that could be construed as a potential conflict of interest.
