# Supplementary material for: Neural correlates of short-term memory in primate auditory cortex
Source: Front Neurosci. 2014 Aug 14;8:250. doi: 10.3389/fnins.2014.00250 (PMC4132374; doi:10.3389/fnins.2014.00250)
Supplement: Supplementary file 1 [file DataSheet1.PDF]

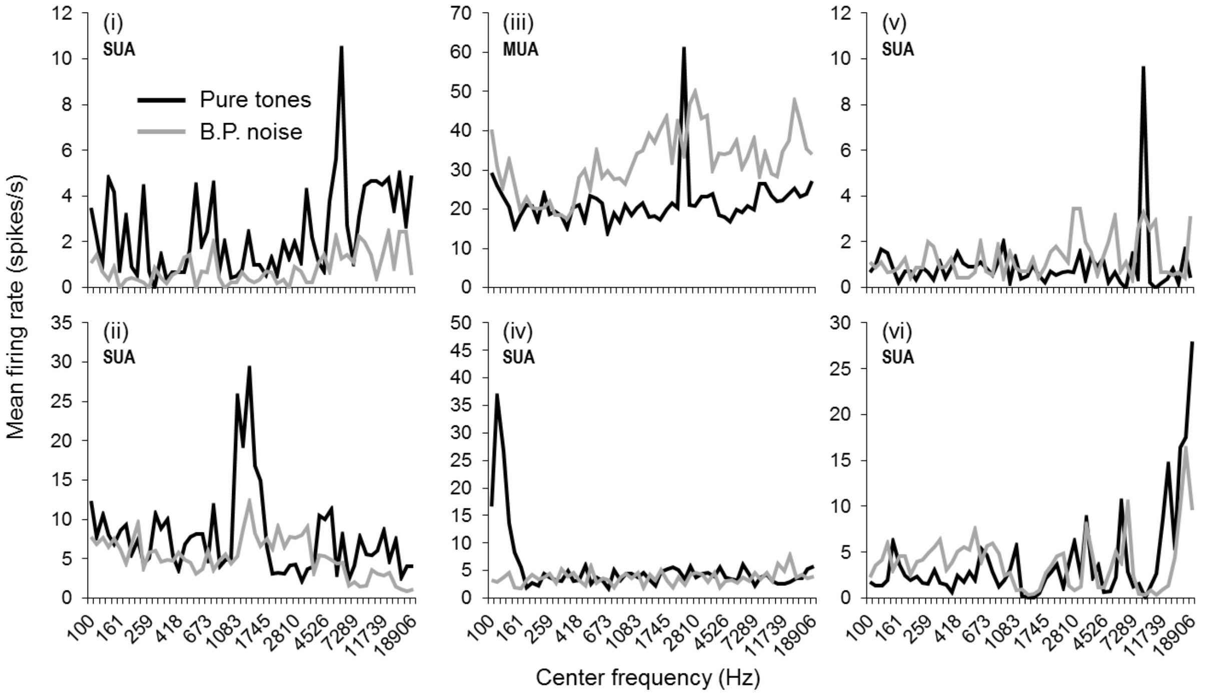

Figure S1 | Tuning profiles for example units assessed by passive presentations of pure tones and band-passed noise. Following the short-term memory task, each unit was passively exposed to a range of 56 pure tones and band-passed noise stimuli with center frequencies spanning .1-18.9 kHz. Each sound was repeated 9-11 times in pseudorandom order separated by a variable interstimulus interval (mean: 1320 ms; range: 1200-1500 ms). The response to each sound was defined as the mean firing rate during the 500-ms sound presentation period.
