# Supplementary material for: Neural correlates of short-term memory in primate auditory cortex
Source: Front Neurosci. 2014 Aug 14;8:250. doi: 10.3389/fnins.2014.00250 (PMC4132374; doi:10.3389/fnins.2014.00250)
Supplement: Supplementary file 2 [file DataSheet2.PDF]

Figure S2 | Spectrograms and temporal envelopes for the sounds presented as memoranda during the short-term memory task. One of the twelve stimulus sets (rows), which each contained one exemplar of each of eight sound types (columns), was pseudorandomly selected for each experimental session. See Methods for additional details.

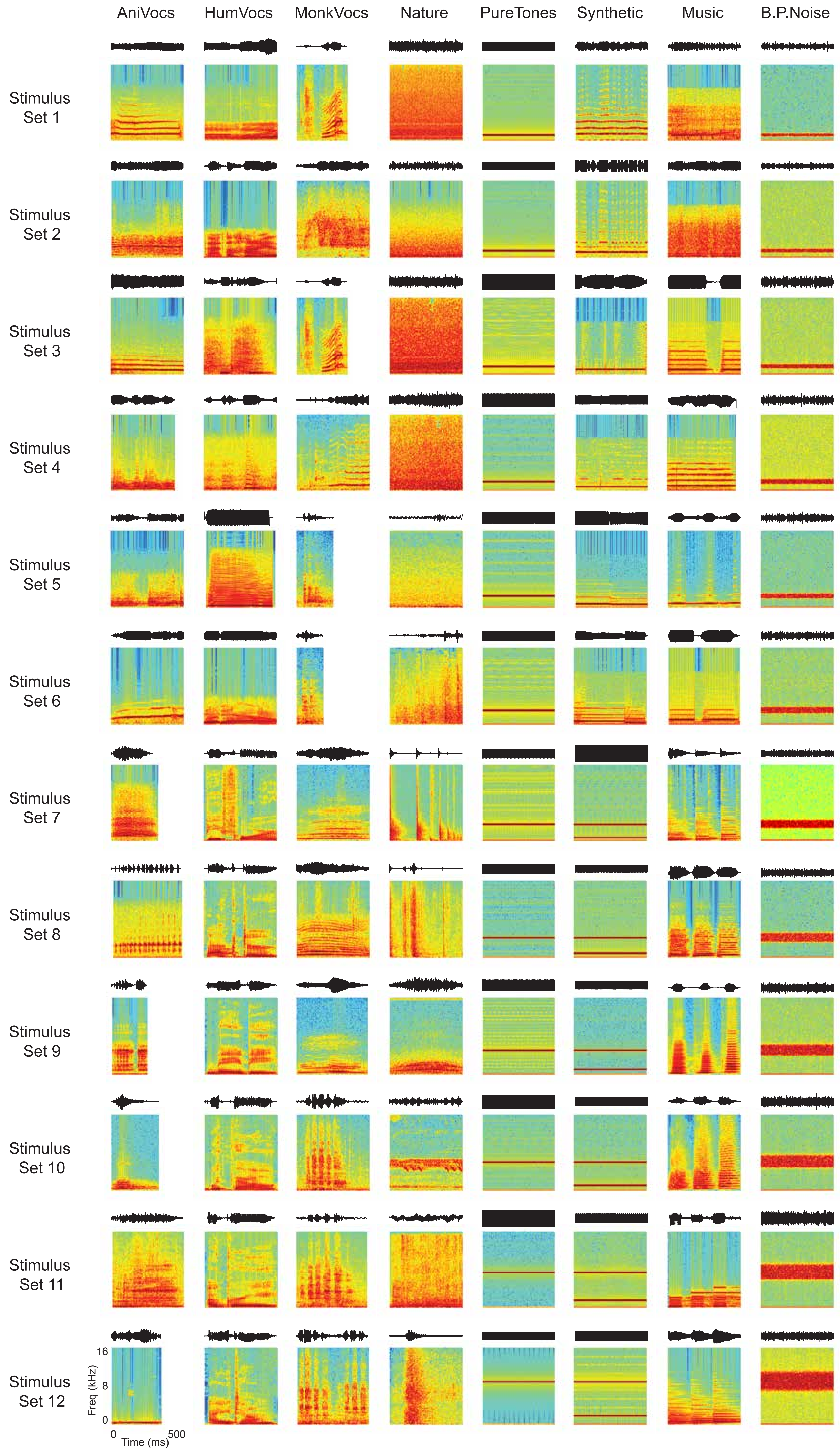
AniVocs
HumVocs
MonkVocs
Nature
PureTones
Synthetic
Music
B.P.Noise
Stimulus Set 1
Stimulus Set 2
Stimulus Set 3
Stimulus Set 4
Stimulus Set 5
Stimulus Set 6
Stimulus Set 7
Stimulus Set 8
Stimulus Set 9
Stimulus Set 10
Stimulus Set 11
Stimulus Set 12
Freq (kHz)
16
8
0
0
Time (ms)
500
